# Rescue of TCA Cycle Dysfunction for Cancer Therapy

**DOI:** 10.3390/jcm8122161

**Published:** 2019-12-06

**Authors:** Jubert Marquez, Jessa Flores, Amy Hyein Kim, Bayalagmaa Nyamaa, Anh Thi Tuyet Nguyen, Nammi Park, Jin Han

**Affiliations:** 1 Department of Health Science and Technology, College of Medicine, Inje University, Busan 47392, Korea; jcuevas.marquez@gmail.com (J.M.); amyhikim@gmail.com (A.H.K.); 2 Department of Physiology, College of Medicine, Inje University, Busan 47392, Korea; jeflores1@up.edu.ph (J.F.); n_bayalgaa@yahoo.com (B.N.); nguyenthituyetanh_t57@hus.edu.vn (A.T.T.N.); 3 Department of Hematology, Mongolian National University of Medical Sciences, Ulaanbaatar 14210, Mongolia; 4 Cardiovascular and Metabolic Disease Center, Paik Hospital, Inje University, Busan 47392, Korea; nammi780314@gmail.com

**Keywords:** mitochondria, TCA, cancer, IDH, SDH, FH, MDH, CRISPR/Cas9, miRNA

## Abstract

Mitochondrion, a maternally hereditary, subcellular organelle, is the site of the tricarboxylic acid (TCA) cycle, electron transport chain (ETC), and oxidative phosphorylation (OXPHOS)—the basic processes of ATP production. Mitochondrial function plays a pivotal role in the development and pathology of different cancers. Disruption in its activity, like mutations in its TCA cycle enzymes, leads to physiological imbalances and metabolic shifts of the cell, which contributes to the progression of cancer. In this review, we explored the different significant mutations in the mitochondrial enzymes participating in the TCA cycle and the diseases, especially cancer types, that these malfunctions are closely associated with. In addition, this paper also discussed the different therapeutic approaches which are currently being developed to address these diseases caused by mitochondrial enzyme malfunction.

## 1. Introduction

Mitochondrion is a maternally hereditary, subcellular organelle which plays a role in bioenergetics, biosynthesis, and cell signaling [1]. The human mitochondrial proteome is composed of a subset of ~20,000 distinct mammalian proteins which are localized in the said organelle. Thirteen of these proteins are encoded by mitochondrial DNA (mtDNA) and the rest are encoded by nuclear DNA (nDNA) [2]. Mutations in these mitochondrial protein genes are notably implicated in diseases such as cancer and diabetes, as well as a plethora of other genetic diseases [3]. Mitochondria have a double lipid membrane with various types of membrane proteins which are divided into four divisions: The intermembrane space (IMS), outer mitochondrial membrane (OMM), inner mitochondrial membrane (IMM), which has a highly particular structure to create cristae of large surface area for ATP production, and the mitochondrial matrix [4]. The mitochondria play a key role in ATP production and circulation based on the availability of energy from calories and oxygen, along with the demands for cellular maintenance and reproduction [3]. Carbon sources from glycolysis, fatty acids, and glutamine are utilized to produce ATP. Carbon sources entering the tricarboxylic acid (TCA) cycle in the mitochondrial matrix produce NADH and FADH_2_, which transfer their electrons to the electron transport chain (ETC) located in the IMM [5]. In the ETC system, the electrons transferred from NADH/FADH_2_ to oxygen induce an oxidation-reduction reaction at each step, and energy from the oxidized electron is utilized to pump protons from the mitochondrial matrix into the intermembrane space through complex I (NADH dehydrogenase), complex III (CoQH2-cytochrome c reductase), and complex IV (cytochrome c oxidase) [6]. The proton gradient is harnessed to drive the switch of ADP to ATP by complex V (ATP synthase), during which concurrently pumped protons return to the matrix. In the matrix, lipids are oxidized by β-oxidation as a breakdown of fatty chains to produce acetyl-CoA [7].

**Figure 1:**
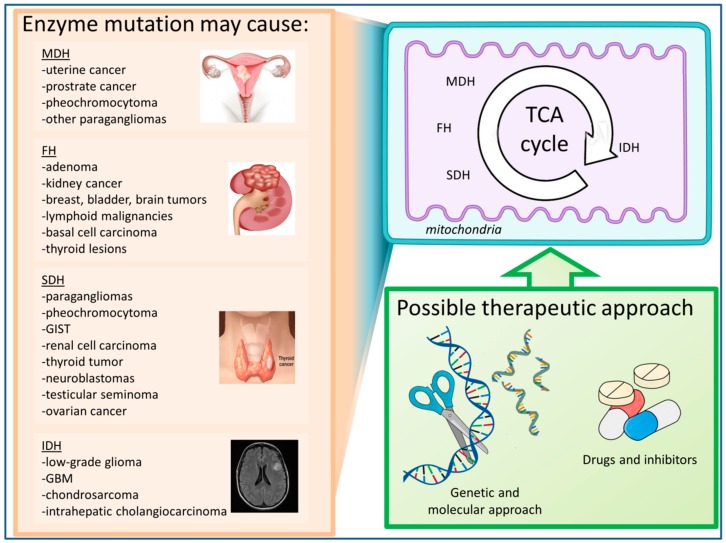
Mutations in the tricarboxylic acid (TCA) cycle enzymes may result in various kinds of cancers. These diseases may possibly be treated through pharmacological and genetic therapeutic approaches. IDH, isocitrate dehydrogenase; SDH, succinate dehydrogenase; FH, fumarate hydratase; MDH, malate dehydrogenase.

Energy production during the metabolic process has recently been in the spotlight due to its capability to generate signaling molecules for various cellular responses. Recent technological developments have further supported long-withstanding hypotheses regarding the key role of aberrant energy production and metabolism in disease models (Figure 1). Therefore, in this review, we discussed the metabolic differences in normal and disease models, and highlighted TCA enzymes and proteins critical in further understanding disease progression along with how we can harness the knowledge regarding these enzymes and proteins in order to address diseases, especially in cancer.

## 2. The TCA Cycle: In Sickness and in Health

The TCA cycle unifies the carbohydrate, lipid, and protein metabolism pathways. In a healthy, normal cell, glycolysis is responsible for the oxidation of the glucose molecule into pyruvate to produce ATP, which is then decarboxylated into acetyl-CoA as it enters the mitochondria, allowing it to enter the TCA cycle. However, each body part exhibits strikingly different metabolic profiles. For example, in the human brain, with the exception of prolonged fasting states, glucose is the main source of energy [8]. Muscles, on the other hand, have a vast reservoir of glycogen that can easily be converted into glucose 6-phosphate [9]. The liver, which is controlled by both neuronal and hormonal systems, provides energy for organs, such as the brain and muscle, in addition to extrahepatic tissues. The liver can produce glucose by breaking down its stored glycogen and through gluconeogenesis. However, in the fasting state, ketone bodies’ conversion from fatty acids is facilitated through mitochondrial β-oxidation and ketogenesis [10,11].

TCA intermediates also play important roles in pathways in which they leave the cycle to be converted into glucose, fatty acids, or non-essential amino acids. Once removed, these intermediates need to be replaced to allow continued function and cycle, known as anaplerosis [12]. In heart and skeletal tissues, anaplerosis maintains steady-state concentrations of TCA intermediates [13]. Cancer cells, on the other hand, transport glucose-derived pyruvate into the mitochondria, where it is used as an anaplerotic substrate to replace TCA intermediates used for biosynthesis [14]. Inadequate amounts of glutamine or suppressed glutaminase forces the cancer cells to depend on glucose carbon flux through pyruvate carboxylase to keep oxaloacetate production and continue downstream TCA cycle activity [15]. In cases like non-small-cell lung carcinoma and glioblastoma, they more frequently rely on pyruvate anaplerosis to maintain TCA cycle flux.

In comparison to the organs previously mentioned, there is an immense demand for energy in mammalian hearts due to its continuous and incessant beating. Central to energy transduction in the heart, the mitochondria generate more than 95% of the ATP used by the heart. In a normal heart, fatty acyl-coenzyme A (CoA) and pyruvate fuel the mitochondria. The entry of long-chain acyl-CoA into the mitochondria is rate-limited by carnitine-palmitoyl transferase-1 (CPT1), while pyruvate dehydrogenase (PDH) reaction regulates pyruvate oxidation. Substrates, such as lactate, ketone bodies, and amino acids, freely enter the mitochondria for oxidation [16].

## 3. TCA Enzymes: The Future to Understanding The Complexities of Diseases

Diseases of the TCA cycle constitute a group of rare human diseases that affect core mitochondrial metabolism [17]. The deficiency of enzymes involving the TCA cycle was detected in order to obtain crucial roles in several human diseases.

Emerging evidences suggest that cancer is mitochondrial in nature [18,19]. Warburg originally observed and postulated that excess lactate production by tumors in the presence of oxygen is a sign of mitochondrial dysfunction, eventually giving rise to the idea of aerobic glycolysis or the ‘Warburg effect’ [20]. Recent studies proved that the dysfunction observed by Warburg is merely an altered state of the mitochondria and is a part of a bigger picture in cancer bioenergetics [21,22,23]. The majority of cancer cells generate most of their ATP through the mitochondria. Few tumors bear TCA enzymes mutations, such as isocitrate dehydrogenase (IDH), succinate dehydrogenase (SDH), fumarate hydratase (FH), and malate dehydrogenase (MDH) [24]. Their mutations are also involved directly or indirectly, which comprises the activation of a hypoxic cellular response and high levels of ROS often found in cancer cells [25].

### 3.1. Isocitrate Dehydrogenase

**Figure 2:**
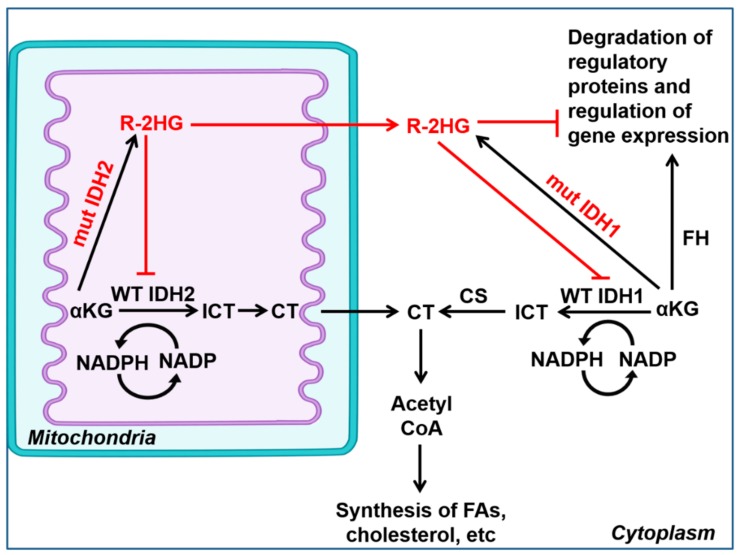
Schematic presentation of the difference in cellular pathways of wild-type and mutated IDH1/2 enzymes during reverse reductive carboxylation reaction. IDH1/2 enzymes catalyzes both the forward and reverse conversion of isocitrate to αKG. Mutations in IDH1/2 cause elevated levels of R-2HG (D-2HG), which is a pro-oncogenic factor. αKG, α-ketoglutarate; ICT, isocitrate; CT, citrate; CS, citrate synthase; FH, fumarate hydratase; FAs, fatty acids. Adapted from Al-Khallaf H. (2017) [26].

**Figure 3:**
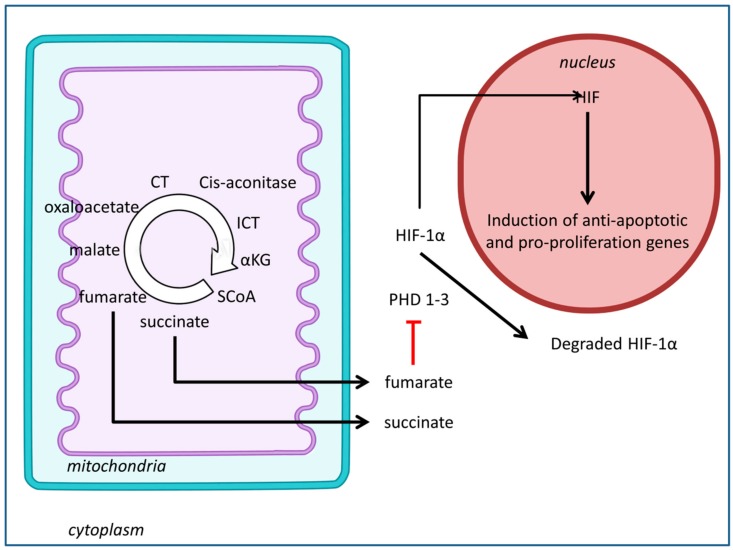
Schematic diagram of tumorigenesis in succinate dehydrogenase (SDH) and fumarate hydratase (FH). SDH and FH deficiency causes accumulation of succinate and fumarate, respectively, inside the mitochondria. These will be subsequently transported into the cytosol. High levels of succinate and fumarate can inhibit prolyl hydroxylases (PDH1-3), which plays a role in the degradation of HIF-1α under normoxic conditions. HIF-1α, when stabilized, induces transcription of nuclear genes involved in tumor suppression. αKG, α-ketoglutarate; ICT, isocitrate; CT, citrate; SCoA, Succinyl-coA; HIF, hypoxia-inducible factor Adapted from Zanssen S, Schon EA (2005) [49] and Shuch, B., Linehan, W.M., & Srinivasan, R. (2013) [50].

Isocitrate dehydrogenase (IDH) is mainly known for its role in catalyzing the oxidative decarboxylation of isocitrate, resulting in 2-oxoglutarate (*α*-KG) and CO_2_ (Figure 2). IDH exists in three isoforms: IDH1 is present in the cytoplasm and peroxisomes, while IDH2 and IDH3 are located in the mitochondrial matrix. IDH1/2 isoforms were also identified to mediate the reverse reductive carboxylation of *α*-KG to isocitrate, which oxidizes NADPH to NADP+. Meanwhile, IDH3 only facilitates the irreversible, NAD-dependent conversion of isocitrate to *α*-KG [26,27]. ODH3 mutation of IDH1/2 is found in low-grade glioma and secondary glioblastoma (GBM), chondrosarcoma, intrahepatic cholangiocarcinomas, hematologic malignancies, premalignant diseases, and rare inherited metabolism disorders [28]. In a clinical study, IDH1 and IDH2 mutations were observed in 16%–17% of patients with AML, in around 20% of angioimmunoblastic T-cell lymphomas (AITL) with worse prognosis [29], and in some low-frequency cancer malignancies [30,31,32,33]. Mutations in the gene-encoding the said enzymes cause increased production of R-2-hydroxyglutarate (R-2HG), an oncogenic factor promoting leukemogenesis (Figure 3) [34,35]. R-2HG produced by mutant IDH in low-grade glioma was shown to activate the mammalian target of rapamycin (mTOR) signaling pathway, which is important for cell growth and metabolism [36]. Moreover, inhibition of the IDH2/R140Q somatic mutant inhibitor induces differentiation of the human erythroleukemic cell line (HEL) and human primary AML cells [37]. Furthermore, mutant IDH induces hypermethylation of *MIRNA148A*, a tumor-suppressive miRNA in glioma CpG island methylator phenotype (G-CIMP) [38].

The most common IDH mutation found in cancer is the substitution of a single arginine in the catalytic site of the enzyme, R132 in IDH1 and R140 or R172 in IDH2, which results in a gain of function. Alterations in R132 in IDH1 and either R172 or R140 in IDH2 represent the majority of IDH mutations identified in cancers [39]. IDH1/R132 and IDH2/R172 are commonly found in gliomas, cholangiocarcinomas, and chondrosarcomas, with a higher frequency of IDH1/R132 mutation occurring in these cancers (58%–90%, 40%–50%, and 50%–60% respectively) compared to IDH2/R172 (3%–5%, 5%–10%, and 10%). These mutations are also found at relatively lower frequencies in AML. Meanwhile, IDH2/R140 is the most common mutation found in AML (30%–50%). However, IDH2/R140, unlike IDH1/R132 and IDH2/R172 mutations, is not found in gliomas, cholangiocarcinomas, and chondrosarcomas [40]. IDH2/R140Q is the most common mutation (75%–80%) and confers a favorable or insignificant impact on overall survival [41,42,43,44,45]. However, IDH2/R172K mutation is found in 20% of the cases, with a lower complete remission rate, higher relapse rate, and lower overall survival [46,47]. Three variants that occur in exon 4 were discovered upon IDH2 gene screening: c.543+45G>A, c. 389 A>T, p. Lys120Met and c.414 T>C, and p.Thr138Thr. These gene variants were found in two independent patients classified under French and Tunisian familial cases which, despite ethnic differences, were similarly diagnosed with non-Hodgkin lymphoma [48]. Melissa Carbonneau et al. found that the molecular mechanism underlying the oncogenic activity of mutant IDH1/2 involved mTOR signaling via KDM4A inhibition, an αKG-dependent enzyme [35].

### 3.2. Succinate Dehydrogenase

Succinate dehydrogenase (SDH) is an enzyme bound to the inner mitochondrial membrane, where it oxidizes succinate to fumarate, and is classified as a tumor suppressor [51]. The SDH complex consists of four subunits (SDHA, SDHB, SDHC, and SDHD), and a deficiency of this enzyme is known to activate tumor formation through dysregulation of HIF activity [52]. HIF, which is a transcription factor, can activate anti-apoptotic and pro-proliferation genes, leading to tumor formation of cancer cells (Figure 3). In addition, deficiency of the enzyme can cause accumulation of the metabolite, succinate. Succinate was shown to exert pro-inflammatory effects through the generation of mtROS [53]. The SDH complex gene-associated cancers include paragangliomas, pheochromocytomas, gastrointestinal stromal tumors (GIST), SDH-deficient renal cell carcinoma [54,55], thyroid tumors, neuroblastomas, testicular seminoma, and ovarian cancer [56].

The SDHA gene is responsible for encoding SDH enzyme major catalytic subunit, possessing a covalently attached flavin adenine dinucleotide (FAD) prosthetic group which can bind with substrates, such as fumarate and succinate, and also with physiological regulators, such as oxaloacetate and ATP. Inactivation of SDHA has been shown to promote neurodegenerative diseases like Leigh syndrome, which is an early-onset encephalopathy [57,58,59], as well as late-onset optic atrophy, ataxia, and myopathy [60]. In addition, a missense mutation in SDHA was shown to cause a multisystemic failure, leading to neonatal death [61]. Meanwhile, in SDH-deficient GISTs, despite the presence of other SDH subunit gene germline mutations, *SDHA* is the most prevalent among the four [62].

SDHB is an enzyme that catalyzes succinate oxidation. SDHB mutations normally lead to extra-adrenal paragangliomas (PGLs), which are usually characterized by highly aggressive tumors, poor prognosis, and early-age onset (~30 years) [63,64]. To a lesser extent, it may also impose risks of adrenal pheochromocytoma (PCC) and head and neck paragangliomas (HNPGLs) [63,64,65,66]. In addition, renal cell carcinoma and T-cell acute leukemia are also associated with SDHB mutations [67,68]. In the study of Fishbein et al., the authors collected and screened data of 173 PGLs/PCCs patients from The Cancer Genome Atlas. *SDHB* appeared to be the most common germline mutation (9%) and exhibited the highest number of copy number alteration (57%) [69].

SDHC mutations were initially implicated with HNPGLs alone. However, recent rare cases of adrenal PCCs and extra-adrenal PGLs were observed to be related to SDHC mutation as well [70,71,72]. Clinically, features of SDHC-associated cases are similar to symptoms of sporadic HNPGLs [73]. In addition, somatic *SDHC* mutations were also detected in 5% of sporadic thyroid cancer cases in a cohort study [74].

SDHD mutations are usually related to multifocal HNPGLs and sometimes to adrenal PCCs and extra-adrenal PGLs, which are usually benign. Metastatic HNPGLs have been described within SDHD mutation carriers with 0%–10% prevalence [70,75]. In the study of Marc Bennedbaek et al., the authors identified 18 different germline variants of SDH in the Danish PGL and PCC patients, wherein 12 were likely pathogenic/pathogenic [76]. Furthermore, PGL/PCC syndrome has also been associated with mutations in SDH assembly factor 2 (SDHAF2) [77,78], which is required for the flavination of SDH [79]. Meanwhile, in sporadic thyroid cancers, about 6% of patients showed germline mutation of *SDHB* or *SDHD* [74].

### 3.3. Fumarate Hydratase

Fumarate hydratase (FH) is responsible for the hydration/dehydration of fumarate to malate, an integral process in cellular respiration and energy production. Similarly to succinate, an increase in fumarate inhibits prolyl hydroxylases, which are responsible for the regulation of HIF-1α degradation. Fumarate upregulation may also cause post-translational modification and inactivation of Kelch-like ECH-associated protein 1 (KEAP1). KEAP1 is a substrate adapter protein for the E3 ubiquitin ligase complex which targets nuclear factor erythroid 2-related factor (NRF2) [80]. NRF2, on the other hand, is a regulator of cellular antioxidant defense [81]. Fumarate have also been proved to bind with glutathione to form the oncometabolite succinate glutathione (GSF), which can act as an alternative substrate to glutathione reductase, thus decreasing NADPH levels and enhancing mt ROS and HIF-1 activation. The aforementioned binding can also cause a further increase in oxidative stress due to the depletion of the antioxidant molecules in the system. Furthermore, in the study by Tyrakis et al., increased fumarate due to the deficiency of FH caused the impairment of the respiratory chain complex 2 via the succination of members of Fe-S cluster biogenesis proteins, which are important for the activity of mitochondrial enzymes [82].

Fumarase gene germline mutation is connected to multiple cutaneous and uterine leiomyomas (MCUL) and hereditary leiomyomatosis and renal cell cancer (HLRCC) [83,84]. Meanwhile, in a study where tissue samples from leiomyosarcoma and uterine leiomyoma patients were analyzed, no somatic mutations in the *fumarate* gene was detected, implying that the somatic mutation in gene-encoding the said enzyme does not play a major role in the development of sporadic leiomyosarcomas or uterine leiomyomas [85]. On the other hand, in the cohort study of PGLs conducted by Letouzé et al., *FH* somatic mutation was detected in the only sample of hypermethylated PGL that did not possess SDHx mutation [86].

An estimated 90% (76%–100%) of families were found to have clinically suggestive HLRCC with predisposed early onset, aggressive form of type 2 papillary renal cell carcinoma [87,88]. FH mutation in kidney cancer has been shown to induce an increase in glucose uptake, glycolytic rate, and contribution of glucose to the pentose phosphate pathway [89]. In another study in clear cell renal cancer, a mutation in FH led to the accumulation of HIF-2α, a promotor renal carcinogenesis [90]. However, Tong. W.H. et al. showed that FH mutations in kidney cancer are associated with a reduction in the activity of the metabolic sensor, AMP-activated protein kinase (AMPK), which leads to increased synthesis of fatty acids and proteins to support ongoing cellular anabolism [91]

### 3.4. Malate Dehydrogenase

Malate dehydrogenase is responsible for the reversible oxidation of malate to oxaloacetate through NAD+ to NADH reduction in the ETC, a critical step in the cellular respiration of cells. However, the role of MDH is not only limited to the ETC, as it also plays important roles in metabolic pathways, including glyoxylate bypass, amino acid synthesis, glucogenesis, and oxidation/reduction balance [92].

The ubiquity of MDH is related to its numerous isoforms, which have different areas of subcellular localization and co-enzyme specificity. In eukaryotic cells, there are two main isoenzymes: The mitochondrial and the cytoplasmic malate dehydrogenase. Mitochondrial malate dehydrogenase (MDH2) is critical in the citric acid cycle, as it catalyzes the reaction of malate to oxaloacetate. The other, cytosolic malate dehydrogenase (MDH1), is a key participant in the malate/aspartate shuttle and catalyzes the conversion of oxaloacetate (OAA) to malate, making transport possible. A third isoenzyme, albeit a minor one, was found in the yeast glyoxysomes, where it catalyzes the malate production from glyoxylate. For comparison, the prokaryotic *Escherichia coli* has only one form and is highly similar in sequence identity and tertiary structure to that of MDH2 [92,93].

Online databases, such as MalaCards, have listed diseases associated with MDH1 to include tetanus neonatorum and x-linked sideroblastic anemia with ataxia. However, a cross-reference has only presented predictability of disease occurrence, and no published literature or data can support the claim. Mutations in the MDH2 gene are related to several cancers, including uterine cancer, prostate cancer, pheochromocytoma, and other paragangliomas. MDH2 is a possible target in cancer therapeutics due to its effect on ATP production and drug sensitivity during knockdown. MDH2 was observed to be overexpressed in doxorubicin-resistant uterine cancer cells and prostate cancer cells and may contribute to drug resistance in disease models [94,95]. Its overexpression could supply more energy for P-glycoprotein in order to flush the chemotherapeutic drugs out, which may account for the shorter periods of relapse-free survival by patients with overexpressed MDH2 after chemotherapy. It is also likely that through the JNK pathway, MDH2 is able to lend docetaxel resistance in prostate cancer cells.

Recently, there has been a lot of interest in the use of potent inhibitors, such as visnagin, which holds potential cardioprotective and anticancer benefits. Researches regarding the inhibition of MDH2 as the only mechanism underlying the visnagin-induced cardioprotection are still in their early stages and require further evaluation.

Extensive research about MDH and its role in cancer is still needed, especially in the clinical aspect. In one recent study, DNA samples from 830 patients with PCCs/PGLs negative for the main PGL driver genes were analyzed. MDH2 variants of unknown significance were interpreted using an algorithm based on 20 computational predictions, enzymatic and immunofluorescence assays, and/or molecular dynamic simulation approach. The researchers identified five MDH2 variants with potential involvement in pathogenicity. Three of these variants were missense mutations and the two remaining ones were an in-frame deletion and a splice-site variant, respectively. All of the mutations were germline and are associated with noradrenergic PCCs/PGLs [96].

## 4. Future Direction of Metabolic Strategies in Combating Diseases Caused by TCA Malfunction

Although common methods such as radiotherapy and chemotherapy exert effectiveness in most patients, they sometimes pose more risks, leading to the development of cardiovascular diseases and eventual progression to heart failure. Any disruption or inefficiency in the metabolic homeostasis can undoubtedly contribute to cardiac pathologies. Such disruptions could stem from factors such as, but not limited to, the inadequate delivery of oxygen and substrates, decreased amounts of high-energy phosphate and the PCR/ATP ratio, and inefficient energy transfer or feedback [97].

When addressing cancer therapeutics, the effects of these strategies should also be considered, since focusing on targets such as enzymes can also prove to be detrimental to the physiological functions of normal cells and tissues. Metabolic inhibitors should minimally, if not entirely, interfere with the patient’s immune system [98]. Nevertheless, new technology has uncovered more novel target pathways that pose less unwanted side effects to the patient.

### 4.1. Inhibitors and Drugs

Multiple preclinical studies have shown IDH as the recent target with the most potential for cancer drugs (Table 1). Preliminary trials indicate the selective inhibitory strength of AG-120 and AG-221 compounds in IDH1 and IDH2 mutant enzymes, respectively, by inhibiting mutant IDH activity and 2-HG accumulation, an oncometabolite [99,100]. AG-120 and another mutant IDH inhibitor, Novartis-530, were found to be the most biochemically potent inhibitors among all nine inhibitors tested in a comparative study [101]. In the same study, both proved to cause the highest reduction of 2-HG levels in six different cancer cell lines with IDH mutation (HT1080 fibrosarcoma, SNU1079, and RBE cholangiocarcinoma, JJ012 chondrosarcoma, U87 glioblastoma, and THP-1 AML). AG-221, on the other hand, significantly improved survival in an IDH2-mutant AML primary xenograft mouse model. Meanwhile, in preliminary phase I clinical trials in patients with advanced hematologic malignancies, the objective response rate ranged from 31% to 40%, with durable responses (>1 year) observed [102]. In addition, AG-881, a brain-penetrant dual IDH1/2 mutant inhibitor, is currently in phase I trial against solid tumors [101].

Inhibition of LDH-A, which facilitates pyruvate conversion to lactate, diminished MYC-driven tumors in xenograft models. In NSCLC mouse models, LDH-A inhibition caused the regression of established tumors without associated toxicity [103,104]. In addition, the genetic ablation of LDH-A has been observed to delay the progression of myeloid leukemia [105]. However, the impact of LDH-A on the adaptive immune system is yet to be explored. Lactate exhibited inhibitory action against cytotoxic T cells. Thus, blocking LDH-A activity may synergize with other immuno-inhibitors to improve host inflammatory T cell activity, which will eventually lead to the targeted tumor cells [106].

Meanwhile, in another clinical trial, dichloroacetate (DCA) was used in patients with lactic acidosis caused by rare inborn errors of mitochondrial metabolism. This small molecule targets pyruvate dehydrogenase kinase (PDK), an enzyme increased in different cancers due to increased activation of hypoxiainducible factor (HIF). PDK negatively regulates the pyruvate dehydrogenase complex (PDH) and blocks the oxidative decarboxylation of pyruvate to acetylCoA, which is important in leading pyruvate into the TCA cycle and away from lactate production [107]. Therefore, PDK inhibition by DCA causes the activation of PDH, enhanced pyruvate to acetyl CoA conversion, and decreased lactate production. More importantly, DCA is well-tolerated by patients even at doses that can affect the mitochondrial membrane potential [108,109].

### 4.2. Novel Approaches to TCA Targeting

However, due to the probable unwanted side effects of drug inhibitors, researchers have turned to revolutionary techniques, such as targeting microRNA (miRNA) and the use of CRISPR/Cas9 to address cancer therapy. Considering current cancer treatment regimens employing DNA-damaging drugs and/or radiation, these new approaches were conceived to be less genotoxic and cause less undesired DNA lesions in cells. This approach also outweighs the ethical limitations of mitochondrial replacement therapy. Here, we concisely discuss miRNA targeting and CRISPR/Cas9 system, both of which are promising candidates in addressing cancer therapies.

### 4.3. miRNA Targeting

MicroRNAs were recently brought to the spotlight due to their key roles in cancer cell metabolism, as multiple pieces of evidence have shown miRNA dysregulation in several types of cancer [113]. MicroRNAs are small, highly evolutionarily conserved, single-stranded, non-coding RNA molecules involved in the regulation of various gene expression. Their regulatory functions are performed through the assembly of RNA-induced silencing complex (RISC), which targets the 3′ untranslated region (UTR) of their respective mRNA [114]. The miRNA serves as a guide for the RISC by base-pairing with the target mRNA, and the level of complementarity between the guide and the target determines the mechanism of silencing: (1) Cleavage of target mRNA with subsequent degradation or (2) translation inhibition [115].

miRNAs can regulate the TCA cycle both directly and indirectly. miRNAs can downregulate subunits of pyruvate dehydrogenase (PDH), the enzyme responsible for the process that bridges glycolysis and TCA cycle—the conversion of pyruvate to acetyl CoA [116]. For example, miR-26a can inhibit the PDH protein X component (PDHX) [110], while miR-146b-5p and miR-370 can downregulate the PDHB subunit [111,112]. On the other hand, glutamine provides a major source of energy for proliferating cancer cells, and glutamine intermediates can be converted to α-KG. miR-137 targets ASC family transporter 2 (ASCT2), a glutamine transporter upregulated in different kinds of cancer. This downregulation of the transporter decreases the level of glutamine metabolism and affects cell survival in colorectal carcinoma, glioblastoma, prostate, and pancreatic cancers [117]. Glutamine metabolism suppression is increasingly being considered as a possible anticancer strategy. Glutaminase inhibitor CB-839 is currently undergoing a phase one clinical trial. CB-839 obstructs glutamine during the glutamate conversion process and changes the pathways of several downstream processes, such as the TCA cycle, glutathione production, and amino acid synthesis [118]. Epigallocatechin gallate (EGCG) reduced tumor growth in preclinical studies by interrupting the anaplerotic use of glutamine in the TCA cycle and is currently undergoing early phase one clinical trials [119].

In addition, miRNAs also play a pivotal role in regulating IDH. miR-183 has been shown to suppress IDH2, which causes a decrease of α-KG levels and a subsequent increase in aerobic glycolysis in glioma cells [120]. Meanwhile, in solid cancer tumors that rely heavily on lipid oxidation for energy source, IDH1, a cytoplasmic isoform of IDH, serves as an important contributor to lipid synthesis. This contribution can be traced back to the role of IDH in converting α-KG to isocitrate, which is subsequently converted to citrate, a precursor for the formation of monounsaturated fatty acids. miR-181a, which targets IDH1, causes a decrease of expression of genes related to lipid synthesis and increases the expression of genes involved in β-oxidation, which subsequently reduces lipid accumulation [121]. Moreover, miR-181a has also shown to sensitize A549 lung cancer cells to cancer drugs by stimulating Bax oligomerization and the activation of proapoptotic caspases [122]. The increased expression of the aforementioned miRNA was also found to increase the sensitivity of mature T cells to peptide antigens. Hence, inhibiting miR-181a in immature T cells not only reduces its sensitivity, but also impairs the positive and negative selection function of immune cells [123].

To take advantage of the significant role of miRNAs as post-transcriptional regulators, several miRNA-based gene therapies were developed for use against cancers. One strategy is to import exogenous tumor suppressor miRNAs, which can either inhibit the tumor cell proliferation or induce apoptosis. These exogenous miRNAs are chemically synthesized mimics of endogenous miRNAs, which are usually downregulated in tumors. These miRNA mimics can be delivered through plasmid DNA or viral vectors. Another strategy is to inhibit the function of oncogenic miRNAs using antisense oligonucleotides. In this strategy, the antagonistic oligonucleotide is complementary to the sequence of the endogenous miRNA and is chemically modified to increase its affinity with the target miRNA. This causes it to be trapped in a configuration that will either result in the inability of RISC processing or degradation of the miRNA itself [124].

The use of miRNA-based gene therapy against cancer is very promising (Table 2). With the advancement of genetic technology, it is highly possible that more miRNA targeting TCA enzymes will be discovered and studied. However, despite its strong logical rationale, there are still problems regarding its logistics and efficiency. miRNA-based therapies specific to TCA cycle enzymes need to be studied further in order to establish its use for TCA enzyme-related cancers.

### 4.4. CRISPR/Cas9 System

Clustered regularly interspaced short palindromic repeats (CRISPR) and CRISPR-associated protein 9, also known as the CRISPR/Cas9 system, was recently found as a potential therapy for cancer due to its gene-editing capability. This was discovered in prokaryotes, which possess CRISPR segments of DNA with short repetitions of base sequences. These sequences are interrupted by spacer sequences, which are remnants of viral or bacteriophage genetic codes, and thus enable recording of DNA sequences that the bacteria have been exposed to [125]. This then strengthens the immune function of the progeny by helping them to detect and to destroy bacteriophages once they attempt to invade the bacteria again. The spacer sequences were transcribed to RNA (crRNA), which recognizes the foreign DNA sequence. The Cas nucleases, on the other hand, mediate DNA cleavage [126]. For use in therapy, the spacer sequences can be genetically modified to recognize mutations in the DNA or predefined sites in the cellular genome and to facilitate cleavage.

Recently, a group of researchers, with the use of this gene-editing tool, designed sgRNAs for 88% of reported cancer mutations [127]. The team envisions this approach as potentially transferable to primary patient samples and for use as a therapeutic approach for personalized treatment. For example, the delivery of Cas9 and mutation-specific sgRNAs into tumor cells byoncolytic viruses could be an efficient technique for targeted therapy. Also, since specific ssgRNAs can be administered together, this strategy could be useful for combination therapy where more than two cancer mutations are targeted at the same time. However, because the method is still in its early stages, the repair mechanism after Cas9-mediated DNA cleavage is limited, resulting in sgRNA-resistant clones or off-target cleavage. In one study, this technique was also used to disrupt the CTCF motif in *IDH* gene in IDH wild-type gliomaspheres. The CTCF insulator protein is an important transcription factor in creating chromatin loops and boundaries that partition topological genome domains. Hypermethylation and/or disruption of binding sites of this transcription factor leads to loss of insulation between topological domains and aberrant gene activation. The aforementioned CRISPR-mediated disruption of the CTCF binding site caused an upregulation of PDGFRA, a prominent glioma oncogene, and increased cell proliferation [128].

In addition to the aforementioned limitations of the approach, several obstacles need to be surpassed in order to effectively use this system, such as possibilities of incomplete editing, inaccurate editing, and off-target mutations [129]. Such inaccuracy of the system may cause inactivation of essential genes, activation of pro-oncotic genes, or rearrangement of chromosomes. The therapy may also impose the risk of causing genetic mosaicism if it fails to affect all cells uniformly. A recent study showed that the genome editing capability of the system is affected by the tumor suppressor p53 [130]. p53 binds to DNA and can stimulate the transcription and activity of p21. p21 will, in turn, interact with cell division-stimulating protein (cdk2), hindering cell division. The CRISPR/Cas9 system has lower efficiency in p53 wild-type cells compared to that of knockout cells. Aside from increasing the efficiency of the CRISPR/Cas9 system, the inhibition of p53 can also decrease the selective advantage of pre-existing p53-deficient/mutant clones, a common characteristic of cancer cells. However, the inhibition of the said tumor suppressor will also increase the risk of cell vulnerability to chromosomal rearrangements and tumorigenic mutations [130].

The CRISPR/Cas9 system is a potentially promising cure for cancer. However, as it is still in its early stages, further research and improvements are needed to ensure its safety. Improving the balance of efficient DNA editing and suppression of potential tumorigenic effects is also important. Considering the ease of its use, its application to other TCA enzymes opens another area for medical research.

## 5. Conclusions

Understanding the mechanisms involving mitochondria is important to further develop strategies in treating mitochondria-associated diseases and dysfunction. The majority of studies to date have focused more on OXPHOS, which is another process that occurs in the mitochondrion and connects the mitochrondion to cancer physiology. However, the TCA cycle is also now being recognized as a key player in certain cancers which involve enzyme dysfunction. This review presented different studies regarding these mitochondrial TCA enzymes and cited diseases where they play a pivotal role. In addition, we also discussed available and prospective treatments, such as the drugs mentioned in the previous section. Last, this review was able to explore two novel approaches which are both promising strategies for cancer treatment: CRISPR/Cas9 and microRNA. However, both strategies require further research to ensure their specificity and efficiency.

## Figures and Tables

**Table 1:** Drugs and inhibitors and their respective TCA or TCA-related enzyme targets.

Drug/Inhibitor	Target	Role of Target	Action of Drug to Target	Sample Type	Reference
AG-120 (Ivosidenib)	IDH1	Catalyze conversion of isocitrate to α-ketoglutarate	inhibit	Clinical trial: glioma, adcanced hematologic malignancy	[87,88]
AG-221	IDH2	Catalyze conversion of isocitrate to α-ketoglutarate	inhibit	Clinical trial: acute myeloid leukemia and myelodysplastic syndrome patients	[86]
Novartis-530	IDH1	Catalyze conversion of isocitrate to α-ketoglutarate	inhibit	Cancer cell lines with somatic IDH1 mutation	[88]
FX 11	LDH-A	Forward and reverse conversion of pyruvate to lactate	inhibit	human lymphoma and pancreatic cancer xenografts	[91]
Dichloroacetate (DCA)	PDK	Phosphorylation and inhibition of PDC	inhibit	Human lung carcinoma cell	[95,96]

**Table 2:** miRNAs and their respective TCA or TCA-related enzyme targets.

miRNA Name	Target	Role of Target	Action of miRNA to Target	Sample Type	Reference
miR-26a	PDHX	Catalyzes conversion of pyruvate to acetyl coA	inhibit	Colorectal cancer cell lines	[110]
miR-146b-5p	PDHB	Conversion of glucose-derived pyruvate to acetyl coA	inhibit	Human colorectal cancer tissue samples, colorectal cancer cell lines	[111]
miR-370	PDHB	Conversion of glucose-derived pyruvate to acetyl coA	inhibit	Human melanoma tissue samples, human melanoma cell line	[112]
miR-137	ASCT2	Transport of glutamine	inhibit	Human neuroblastoma cell line	[107]
miR-183	IDH2	Catalyze conversion of isocitrate to α-ketoglutarate	inhibit	Glioblastoma cell lines	[108]
miR-181a	IDH1	Catalyze conversion of isocitrate to α-ketoglutarate	inhibit	Tail-tip fibroblast, mouse embryonic fibroblast	[109]
				Human lung cancer cell line, human colon cancer cell line, human cervical cancer cell line	[110]
				Mouse T-cells	[111]
